# Dietary Habits Had No Relationship with Adolescent Idiopathic Scoliosis: Analysis Utilizing Quantitative Data about Dietary Intakes

**DOI:** 10.3390/nu11102327

**Published:** 2019-10-01

**Authors:** Keiko Asakura, Takehiro Michikawa, Masashi Takaso, Shohei Minami, Shigeru Soshi, Takashi Tsuji, Eijiro Okada, Katsumi Abe, Masamichi Takahashi, Morio Matsumoto, Yuji Nishiwaki, Kota Watanabe

**Affiliations:** 1 Department of Environmental and Occupational Health, School of Medicine, Toho University, 5-21-16, Omori-Nishi, Ota-ku, Tokyo 143-8540, Japan; JZF01334@nifty.ne.jp (K.A.); takehiro.michikawa@med.toho-u.ac.jp (T.M.); yuuji.nishiwaki@med.toho-u.ac.jp (Y.N.); 2 Department of Orthopedic Surgery, Kitasato University, 1-15-1, Kitasato, Minami, Sagamihara 252-0375, Japan; mtakaso@kitasato-u.ac.jp; 3 Department of Orthopedic Surgery, Seirei Sakura Citizen Hospital, 2-36-2, Eharadai, Sakura-ku, Chiba 285-0825, Japan; minami@sis.seirei.or.jp; 4 Department of Orthopedic Surgery, the Jikei University School of Medicine, 3-19-18, Nishi-Shimbashi, Minato-ku, Tokyo 105-0003, Japan; s-soshi@jikei.ac.jp; 5 Department of Orthopedic Surgery, Fujita Health University, 1-98, Dengakugakubo, Kutsukake, Toyoake 470-1192, Japan; tsuji9@gmail.com; 6 Department of Orthopedic Surgery, Keio University School of Medicine, 35, Shinanomachi, Shinjuku-ku, Tokyo 160-8582, Japan; eijiro888@gmail.com (E.O.); morio@a5.keio.jp (M.M.); 7 The Tokyo Health Service Association, 1-2, Sunatoharacho, Shinjuku-ku, Tokyo 162-0842, Japan; k.abe@yobouigaku-tokyo.jp (K.A.); m.takahashi@yobouigaku-tokyo.jp (M.T.)

**Keywords:** adolescent idiopathic scoliosis, Cobb angle, dietary habits, nutrient, food, junior high school girls

## Abstract

Although several genetic and environmental factors have been identified as risk factors of adolescent idiopathic scoliosis (AIS), the influence of dietary intake has not been elucidated. We evaluated the association between AIS and dietary habits among female students. Junior high school girls aged 12 to 15 years in the Tokyo metropolitan area who underwent a second school screening for scoliosis were recruited. AIS was diagnosed by orthopedic surgeons specializing in scoliosis, using standing whole spine radiography. Students with a Cobb angle of ≥15° were classified into the AIS group, and others were considered healthy controls. Dietary assessment was performed using a validated diet history questionnaire. Dietary intakes were categorized into quintiles based on distribution, and crude and multivariable odds ratios and 95% confidence intervals for AIS for each quintile category of dietary variable were calculated, with the lowest quintile category used as a reference. In total, 2431 subjects were included in the analysis, and 47.8% of them were diagnosed with AIS. None of the selected nutrients or food groups was significantly associated with AIS. In conclusion, dietary habits may not be associated with AIS.

## 1. Introduction

Adolescent idiopathic scoliosis (AIS) is a three-dimensional deformity of the spine, defined as a lateral curvature of the spine in the coronal plane, which develops in children aged >10 years without known causes [1]. Several genetic and environmental factors have already been identified as risk factors of AIS [2,3,4,5,6,7,8,9,10,11,12]. Nevertheless, given this multifactorial background, a full understanding of the factors associated with AIS requires further evaluation. In particular, the influence of dietary intake has not been elucidated. The high AIS concordance rate in monozygotic twins implies the importance of genetic background [2,3,4,5,6], but other studies have indicated that onset is also influenced by environmental factors such as participation in sport activities [7,8,9,10,11,12].

To our knowledge, no epidemiological study has yet investigated the influence of dietary factors on AIS. Some studies reported that bone mineral density in AIS patients was low, and that low calcium intake and vitamin D receptor polymorphism were related with low bone mass in AIS patients [13,14,15]. However, the pathological mechanism between the onset of AIS and calcium/vitamin D intake has not been elucidated. Although other experimental studies in animals suggested that deficiencies of vitamins (e.g., A, B1) and minerals (e.g., copper, manganese) could cause spinal deformity [16,17,18], the effect of these nutrients on AIS has not been investigated.

Recently, our group investigated the relationship between various lifestyle factors and AIS and reported associations with classic ballet training, family history of scoliosis, and low body mass index [19]. That survey included a quantitative assessment of dietary intake using a brief-type self-administered diet history questionnaire for school children and adolescents (BDHQ15y) [20]. Here, we used this data to evaluate the influence of nutrient and food intakes on AIS.

## 2. Materials and Methods

### 2.1. Study Design and Participants

Details of the study design and participant characteristics have been reported in our previous study [19]. Briefly, the present study was conducted under a cross-sectional design in Japanese female junior high school students in the Tokyo metropolitan area who visited the Tokyo Health Service Association (Tokyo, Japan) for a second school screening for scoliosis. In Japan, students in elementary and junior high school are screened for scoliosis at 10 to 14 years of age at school. If scoliosis is suspected, the student is referred to a second screening where a diagnosis is made using whole-spine radiography. The Tokyo Health Service Association is a public service corporation that provides such second screening as well as a variety of other early-detection medical examinations.

Of the students suspected to have scoliosis after the initial screening using Moire images, 76.9% visited the Tokyo Health Service Association for their second screening and were evaluated for scoliosis with standing posteroanterior whole-spine radiographs. Between January 2013 and February 2015, 2759 female students underwent the second scoliosis screening, of whom 2747 (99.6%) participated in this study. None of these students had previously been diagnosed with scoliosis. After excluding students with congenital vertebral anomalies (*n* = 36), congenital heart disease (*n* = 20), epilepsy (*n* = 27), or spina bifida (*n* = 1), those whose menarcheal status was unknown (*n* = 63), and those with no or insufficient information about dietary intake (*n* = 169), the remaining 2431 students were included in our analyses. This study was approved by the ethics committees of the authors’ institutions. Students and their guardians were given a pamphlet describing the study, a brief oral explanation, and a questionnaire. Those who completed the questionnaire were deemed to have consented to participation in this study.

### 2.2. Questionnaire

The students and their guardians completed two questionnaires while waiting for the standing posteroanterior radiographs of the spine and, therefore, before receiving any results from the radiographs or recommendations for further follow-up or treatment to avoid reporting bias. The first questionnaire asked about lifestyle, physical activity, home environment, family history of AIS, and so on [19]. The second was BDHQ15y, which is used to assess habitual dietary intake during the one month preceding the implementation of the survey. The BDHQ15y is a four-page questionnaire developed based on the self-administered diet history questionnaire (16-page comprehensive type) [21,22,23] and the brief-type, self-administered diet history questionnaire (BDHQ) for adults [21,22]. It can be used for school children and adolescents aged 6–18 years. The basic structure of the BDHQ15y is closely similar to that of the BDHQ for adults but excludes one section about alcoholic beverages. Estimates of daily intake for foods (67 items in total), energy, and selected nutrients were calculated using an ad hoc computer algorithm for the BDHQ15y based on the Standard Tables of Food Composition in Japan [24]. The validity of the BDHQ15y for selected fatty acids and carotenoids using biomarkers (erythrocyte fatty acids and serum carotenoids) as an objective standard has been reported elsewhere [20].

### 2.3. Radiographic Measurements

Standing posteroanterior radiographs of the whole spine were evaluated by orthopedic surgeons specializing in the treatment of scoliosis. Multiple surgeons took part in the evaluation, but one surgeon did the measurement for one participant. The Cobb angle is defined as an angle of curvature be measured by drawing lines parallel to the superior endplate of a proximal-end, most-tilted vertebra and the inferior endplate of a distal-end, most-tilted vertebra of the structural curve. The Cobb angles of the major curves were measured if spinal curvature was present, and AIS was defined as a Cobb angle of ≥15°. The Cobb angles were measured using the DICOM (Digital Imaging and Communications in Medicine) POP (Post Office Protocol)-net server system (ImageONE, Tokyo, Japan). The present study met established standards for the interobserver reliability of the measurements [25]. The Risser sign (grade 0 to 5) was recorded for each participant as an indicator of bone maturity [26]. Any congenital anomalies of the vertebrae were also recorded for later exclusion from the analysis.

### 2.4. Statistical Analysis

To select subjects with appropriate dietary intake data, we eliminated subjects who had not answered BDHQ15y and those whose energy intake estimated using the BDHQ15y was not between 0.5 times the estimated energy requirement (EER) for girls aged 12–14 years with the lowest physical activity level (EER I) [27] and <1.5 times the EER for those with the highest physical activity level (EER III) [27] (i.e., those whose energy intake was <1075 kcal or ≥3825 kcal: 169 subjects were eliminated).

Nutrients for analysis were selected based on the physiology of bone [28] and the results of past studies [13,14,15,16,17,18]. Selected nutrients were protein, calcium, vitamins (D, A, K, B6), and minerals (manganese and copper). Foods which abundantly contain the selected nutrients and are frequently consumed in daily life were also selected for the analysis: rice and other cereals, pulses, fruits, vegetables (w/o mushrooms), mushrooms, fish and shellfish, meat, dairy products, and tea and coffee. Food groups were defined based on the categories in the food composition tables of Japan [24]. Because intakes of most nutrients were positively associated with energy intake, energy-adjusted intakes were calculated by the density method [29]. The amount of each nutrient consumed daily was calculated as a percentage of daily energy intake (% energy) for energy-containing nutrients (i.e., protein) or weight (g, mg, or μg) per 1000 kcal of daily energy intake for non-energy-containing nutrients. The intakes of each food group were energy-adjusted in the same manner and presented in weight (g) per 1000 kcal of energy intake.

Odds ratios (ORs) for AIS in relation to dietary intakes were estimated by logistic regression analysis. We categorized dietary intakes into quintiles based on distribution, and calculated crude and multivariable ORs and 95% confidence intervals for AIS for each quintile category of dietary variable, with the lowest quintile category used as a reference. Multivariate-adjusted odds ratios were calculated by adjusting for potential confounding factors including age, menarcheal status, school type (public or private; as a surrogate marker of household income), and family composition (single-parent family, 2-parent family, or 3-generation family) as our previous study [19]. In addition, a second model included these factors plus classic ballet experience (yes or no), mother’s history of scoliosis (yes or no), use of dental braces (yes or no), and BMI (<18.5, 18.5 ≤ 25, 25≤). These new covariates were selected based on their significant association with AIS in our previous study [19]. Trends of association were tested using logistic regression models, which assigned scores at the level of the independent variable (dietary intake). All analyses were performed with Statistical Analysis System (SAS) version 9.4 software (SAS Institute, Cary, NC, USA). Statistical tests were two-sided, and *p* values of <0.05 were considered statistically significant.

## 3. Results

Characteristics of the participants are shown in Table 1. In total, 47.8% of the participants had a Cobb angle of ≥15° and were diagnosed with AIS. The mean Cobb angle of the scoliosis group was 20.7 ± 5.6°. The mean age of the control group was 14.0 ± 0.9 years and that in the scoliosis group was 13.7 ± 0.9 years. BMI in nearly half (47.6%) of the participants was less than 18.5 kg/m^2^. The mean BMI in the control group was 19.1 ± 2.3 kg/m^2^ and that in the scoliosis group was 18.5 ± 2.2 kg/m^2^.

The relationship between nutrient intake and AIS is summarized in Table 2. The analyzed subjects were divided into five groups by quintiles of nutrient intakes, and crude and adjusted ORs for AIS were calculated, with the lowest quintile category used as a reference. None of the selected nutrients, including calcium and vitamin D, was significantly associated with AIS. No *p* value for trend showed a dose–response relationship between nutrient intake and AIS. Similarly, the relationship between food groups and AIS is presented in Table 3. There was no significant relationship between food groups and AIS. A *p*-value for trend for pulses by the crude model was 0.07, but multivariate-adjusted ORs were closer to one than the crude OR. No change in results was seen when AIS was defined as a Cobb angle of ≥20°.

## 4. Discussion

This is the first epidemiological study to show no relationship between AIS and dietary habits in adolescents. In the present study, AIS was diagnosed by orthopedic surgeons who specialized in the treatment of scoliosis, and dietary intake was quantitatively assessed with a validated diet history questionnaire at both the nutrient and food levels. Non-significant relationship between food intakes and AIS might have implied that possible combinations of nutrients were not associated with AIS, either. Although it is difficult to confirm the absence of a relationship by statistical testing, the number of subjects in this study was large enough to mention the non-significance of the differences.

Studies of nutrients affecting spinal development and morphology are scarce, particularly those in humans. There have been a few experimental studies in animals [16,17,18]. Li et al. reported that vitamin A deficiency in pregnant rats induced congenital scoliosis in neonates through a defect in retinoic signaling pathway during somitogenesis [16]. Incidence in the vitamin A deficiency group was 13.8% (8/58), while no filial rats in the control group showed abnormalities of the spine. Opsahl et al. showed that the copper content of the diet was significantly associated with onset and severity of spinal lesions in a line of chickens susceptible to scoliosis [17]. Lower copper intake was associated with a greater curvature angle. In addition to copper, deficiencies in vitamin B6 and manganese were also associated with the development of scoliosis in the same kind of chicken [18]. The authors of these studies speculated that deficiency of copper, vitamin B6, and manganese might be associated with abnormal formation of connective tissue components such as collagen [17,18]. Although we assessed these nutrients in the present study, none showed a significant association with AIS.

Regarding humans, Goździalska et al. demonstrated that serum levels of 25-OH-vitamin D_3_ and calcitonin were lower in girls with AIS than in healthy girls [30]. They pointed out the possible involvement of vitamin D deficiency in AIS etiology, but their study did not include dietary assessment. Given other findings that polymorphism in the vitamin D receptor is possibly associated with bone mineral density at the lumber spine [15], which aspect of vitamin D is associated with AIS should be confirmed: intake, absorption, metabolism, or internal function. We saw no relationship between vitamin D intake and AIS in our present study. Since we did not have any information about serum vitamin D concentration as well as ultraviolet light exposure, relationship between vitamin D insufficiency and AIS should be investigated in future studies. The low bone mass in AIS patients is well-known [31] as is its association with low calcium intake. However, calcium intake did not differ between AIS patients and healthy controls in two studies [13,14]. Our present finding supports this lack of association between calcium intake and AIS.

We and others have already reported that low body mass index was associated with a higher prevalence of AIS in female junior high school students [19,32,33]. Given that low BMI implies habitual lower energy intake against energy requirements [34], energy intake might be a dietary factor which is associated with AIS. Further studies are needed to confirm whether more energy intake is effective in preventing the occurrence and progression of AIS.

The results shown in this study may be applied to many developed countries and regions due to the following reasons. It has been reported that the overall prevalence in Japanese schoolchildren 11–14 years of age with Cobb angles of 10° or more was 0.87%, and the prevalence of curves of 20° or more was 0.31% [35]. This prevalence was almost comparable to the global one [36]. Regarding nutrient intakes in the study participants, calcium insufficiency was prevalent (median intake 319 mg/1000 kcal), and some parts of the participants may have suffered from vitamin A (308 μgRAE/1000 kcal) and/or vitamin B6 (0.57 mg/1000 kcal) insufficiency, in consideration of the indices in the Dietary Reference Intakes for Japanese, 2015 [27]. However, symptomatic nutrient deficiencies are rare in Japan, and this situation is similar to those in other developed countries. From a different point of view, we could not find any association between nutrients and AIS which had been observed in animal experiments because the degree of nutrition deficiency in the experiments might have been much severer than the nutrient insufficiency in humans.

Our study has several strengths. The large number of subjects (*n* = 2431) and high response rate (99.6%) supported the high power of the statistical analysis and minimal selection bias. AIS was appropriately diagnosed, and a validated questionnaire (BDHQ15y) was used [20,21,22,23]. Several lifestyle factors have been already assessed as possible risk factors of AIS [19] and were considered possible confounding factors in the present study.

Several limitations of this study also warrant mention. First, all of the subjects were suspected of having scoliosis as a result of their first screening tests before the questionnaire survey. This might have caused a change in attitude to responding, which differed from general population. However, as both the AIS and control groups consisted of subjects who underwent the second screening before they learnt of the result, the comparison should have been performed appropriately. Second, the dietary intakes measured by BDHQ15y are habitual intakes during the one month preceding the implementation of the survey. In other words, we did not evaluate cumulative intake from birth or infancy. Give studies showing the tracking of dietary habits over years [37,38], recent dietary intake may reflect cumulative intake, to some extent at least. Third, due to the cross-sectional nature of the preset study, the causal direction between AIS and dietary factors was unclear—some subjects might have changed their dietary habits after learning of the possibility of AIS. However, this change could have occurred in both the AIS and control groups, because all subjects were suspected of having AIS after the first screening.

## 5. Conclusions

This study showed that nutrient and food intake was not associated with AIS prevalence in junior high school girls in Japan. Influence of energy intake, which was implied by a relationship between low BMI and AIS, might be the next issue warranting investigation.

## Figures and Tables

**Table 1:** Characteristics of the participants.

Variable	n (%) or Mean, SD					
	AIS Group ^1^ (*n* = 1161)		Control Group ^1^ (*n* = 1270)		Total (*n* = 2431)	
Respondent of the questionnaire						
Mother	927	(81.1)	1038	(82.9)	1965	(82.1)
Father	130	(11.4)	122	(9.7)	252	(10.5)
Grandparent	9	(0.8)	14	(1.1)	23	(1.0)
Other	77	(6.7)	78	(6.2)	155	(6.5)
Age (years)						
mean, SD	13.7	0.9	14.0	0.9	13.9	0.9
12	120	(10.3)	84	(6.6)	204	(8.4)
13	379	(32.6)	242	(19.1)	621	(25.6)
14	403	(34.7)	478	(37.6)	881	(36.2)
15	259	(22.3)	466	(36.7)	725	(29.8)
Height (cm)	156.4	5.3	157.5	5.2	157.0	5.3
Weight (kg)	45.3	6.3	47.5	6.7	46.5	6.6
BMI						
mean, SD	18.5	2.2	19.1	2.3	18.8	2.3
<18.5	629	(54.2)	527	(41.5)	1156	(47.6)
18.5 ≤ 25	518	(44.6)	724	(57.0)	1242	(51.1)
≥25	14	(1.2)	19	(1.5)	33	(1.4)
Experienced menarche						
No	122	(10.5)	79	(6.2)	201	(8.3)
Yes	1039	(89.5)	1191	(93.8)	2230	(91.7)
Risser grade						
0–3	428	(38.7)	297	(25.1)	725	(31.7)
4–5	677	(61.3)	887	(74.9)	1564	(68.3)
Mother’s history of scoliosis						
No	1080	(93.0)	1210	(95.3)	2290	(94.2)
Yes	81	(7.0)	60	(4.7)	141	(5.8)
Dental braces use						
No	327	(28.2)	303	(24.1)	1789	(74.0)
Yes	832	(71.8)	957	(76.0)	630	(26.0)
Energy intake (kcal)	2016	533	2032	541	2024	537
Cobb angle (°)						
mean, SD	20.7	5.6	8.2	4.9	14.1	8.1
<15°	0	(0.0)	1270	(100.0)	1270	(52.2)
≥15°, <20°	621	(53.5)	0	(0.0)	621	(25.6)
≥20°	540	(46.5)	0	(0.0)	540	(22.2)
Social factors						
School						
Public	919	(79.2)	1059	(83.7)	1978	(81.6)
Private	241	(20.8)	206	(16.3)	447	(18.4)
Family composition						
Single-parent family	130	(11.2)	166	(13.1)	296	(12.2)
Two-parent family	879	(75.7)	927	(73.0)	1806	(74.3)
Three-generation family	152	(13.1)	177	(13.9)	329	(13.5)
Maternal education (years)						
<10	26	(2.3)	23	(1.9)	49	(2.1)
10–12	350	(31.2)	431	(35.2)	781	(33.3)
13–16	718	(64.0)	742	(60.5)	1460	(62.2)
≥17	28	(2.5)	30	(2.5)	58	(2.5)
Paternal education (years)						
<10	34	(3.1)	52	(4.4)	86	(3.8)
10–12	275	(25.3)	333	(28.3)	608	(26.8)
13–16	671	(61.7)	693	(58.9)	1364	(60.2)
≥17	108	(9.9)	99	(8.4)	207	(9.1)
Classic ballet experience						
No	983	(84.7)	1122	(88.4)	2105	(86.6)
Yes	178	(15.3)	148	(11.7)	326	(13.4)

^1^ AIS group included participants whose Cobb angle was ≥15°. Other participants were included in the control group.

**Table 2:** Multivariate-adjusted odds ratios and 95% confidence intervals for adolescent idiopathic scoliosis by quintile of nutrient intake in 2431 Japanese female junior high school students.

Nutrient	Intake	Quintile Category of Nutrient Intake					p for Trend ^4^
	Median [IQR]	1 (Lowest, *n* = 486)	2 (*n* = 486)	3 (*n* = 487)	4 (*n* = 486)	5 (Highest, *n* = 486)	
Protein (% energy)	13.8 [12.4–15.3]						
n with/without AIS ^1^		231/255	240/246	241/246	226/260	223/263	
Crude OR (95% CI)		ref	1.08 (0.84, 1.39)	1.08 (0.84, 1.39)	0.96 (0.75, 1.24)	0.94 (0.73, 1.21)	0.39
Adjusted OR (95% CI) ^2^		ref	1.03 (0.80, 1.33)	1.06 (0.82, 1.37)	0.92 (0.71, 1.19)	0.94 (0.72, 1.21)	0.41
Adjusted OR 2 (95% CI) ^3^		ref	1.07 (0.83, 1.39)	1.06 (0.82, 1.38)	0.93 (0.72, 1.21)	0.95 (0.73, 1.23)	0.41
Calcium (mg/1000 kcal)	319 [262–393]						
n with/without AIS		227/259	243/243	219/268	240/246	232/254	
Crude OR (95% CI)		ref	1.14 (0.89, 1.47)	0.93 (0.72, 1.20)	1.11 (0.87, 1.43)	1.04 (0.81, 1.34)	0.84
Adjusted OR (95% CI)		ref	1.15 (0.89, 1.48)	0.90 (0.70, 1.17)	1.10 (0.85, 1.43)	1.04 (0.81, 1.35)	0.89
Adjusted OR 2 (95% CI)		ref	1.13 (0.88, 1.47)	0.91 (0.70, 1.18)	1.11 (0.85, 1.43)	1.03 (0.79, 1.33)	0.92
Vitamin D (μg/1000 kcal)	4.6 [3.2–6.3]						
n with/without AIS		222/264	250/236	223/264	223/263	243/243	
Crude OR (95% CI)		ref	1.26 (0.98, 1.62)	1.01 (0.78, 1.29)	1.01 (0.78, 1.30)	1.19 (0.92, 1.53)	0.67
Adjusted OR (95% CI)		ref	1.20 (0.92, 1.55)	0.97 (0.75, 1.25)	0.95 (0.74, 1.23)	1.17 (0.90, 1.51)	0.77
Adjusted OR 2 (95% CI)		ref	1.16 (0.89, 1.50)	0.93 (0.72, 1.20)	0.93 (0.72, 1.20)	1.15 (0.89, 1.49)	0.86
Vitamin A (μgRAE/1000 kcal)	308 [240–414]						
n with/without AIS		228/258	224/262	232/255	223/263	254/232	
Crude OR (95% CI)		ref	0.97 (0.75, 1.25)	1.03 (0.80, 1.32)	0.96 (0.75, 1.24)	1.24 (0.96, 1.59)	0.14
Adjusted OR (95% CI)		ref	0.93 (0.72, 1.20)	0.99 (0.76, 1.28)	0.91 (0.71, 1.18)	1.18 (0.91, 1.53)	0.27
Adjusted OR 2 (95% CI)		ref	0.93 (0.72, 1.21)	0.99 (0.76, 1.29)	0.90 (0.69, 1.16)	1.17 (0.90, 1.52)	0.36
Vitamin K (μg/1000 kcal)	120 [84.8–169]						
n with/without AIS		222/264	217/269	246/241	246/240	230/256	
Crude OR (95% CI)		ref	0.96 (0.75, 1.24)	1.21 (0.94, 1.56)	1.22 (0.95, 1.57)	1.07 (0.83, 1.38)	0.20
Adjusted OR (95% CI)		ref	0.94 (0.73, 1.22)	1.23 (0.95, 1.59)	1.22 (0.95, 1.58)	1.07 (0.83, 1.39)	0.17
Adjusted OR 2 (95% CI)		ref	0.94 (0.73, 1.22)	1.24 (0.95, 1.60)	1.25 (0.96, 1.62)	1.05 (0.81, 1.36)	0.21
Vitamin B6 (mg/1000 kcal)	0.57 [0.50–0.67]						
n with/without AIS		237/249	211/275	239/248	244/242	230/256	
Crude OR (95% CI)		ref	0.81 (0.63, 1.04)	1.01 (0.79, 1.30)	1.06 (0.82, 1.36)	0.94 (0.73, 1.21)	0.59
Adjusted OR (95% CI)		ref	0.79 (0.61, 1.03)	1.02 (0.79, 1.32)	1.08 (0.83, 1.40)	0.93 (0.72, 1.20)	0.59
Adjusted OR 2 (95% CI)		ref	0.79 (0.61, 1.03)	1.02 (0.78, 1.32)	1.06 (0.82, 1.38)	0.91 (0.70, 1.19)	0.70
Manganese (mg/1000 kcal)	1.74 [1.36–2.13]						
n with/without AIS		230/256	237/249	239/248	230/256	225/261	
Crude OR (95% CI)		ref	1.06 (0.82, 1.36)	1.07 (0.83, 1.38)	1.00 (0.78, 1.29)	0.96 (0.75, 1.24)	0.63
Adjusted OR (95% CI)		ref	1.04 (0.81, 1.35)	1.02 (0.79, 1.32)	0.96 (0.75, 1.25)	0.95 (0.73, 1.23)	0.53
Adjusted OR 2 (95% CI)		ref	1.03 (0.80, 1.34)	1.04 (0.80, 1.35)	0.96 (0.74, 1.24)	0.96 (0.74, 1.25)	0.61
Copper (mg/1000 kcal)	0.58 [0.52–0.63]						
n with/without AIS		229/257	229/257	234/253	240/246	229/257	
Crude OR (95% CI)		ref	1.00 (0.78, 1.29)	1.04 (0.81, 1.34)	1.10 (0.85, 1.41)	1.00 (0.78, 1.29)	0.75
Adjusted OR (95% CI)		ref	0.98 (0.76, 1.26)	1.05 (0.81, 1.35)	1.08 (0.84, 1.40)	1.03 (0.80, 1.34)	0.58
Adjusted OR 2 (95% CI)		ref	0.96 (0.74, 1.24)	1.03 (0.80, 1.34)	1.08 (0.83, 1.40)	1.03 (0.79, 1.34)	0.57

IQR, interquartile range; OR, odds ratio; CI, confidence interval. ^1^ AIS was defined as a Cobb angle of ≥15°. ^2^ Adjusted OR was calculated by logistic regression models including age, menarcheal status, school type (public or private), and family composition (single-parent, 2-parent, or 3-generation family) as covariates. ^3^ Adjusted OR 2 was calculated by logistic regression models including classic ballet experience (yes or no), mothers’ history of scoliosis (yes or no), use of dental braces (yes or no), and BMI (<18.5, 18.5 ≤ 25, 25≤), as well as the four covariates mentioned above (2). ^4^ Trends of association were tested using logistic regression models, which assigned scores at the level of the independent variable (nutrient intake).

**Table 3:** Multivariate-adjusted odds ratios and 95% confidence intervals for adolescent idiopathic scoliosis by quintile of food group intake in 2431 Japanese female junior high school students.

Food Group (g/1000 kcal)	Intake	Quintile Category of Food Intake					*p* for Trend ^4^
	Median [IQR]	1 (Lowest, *n* = 486)	2 (*n* = 486)	3 (*n* = 487)	4 (*n* = 486)	5 (Highest, *n* = 486)	
Rice and other cereals	204 [163–248]						
n with/without AIS ^1^		249/237	215/271	233/254	235/251	229/257	
Crude OR (95% CI)		ref	0.76 (0.59, 0.97)	0.87 (0.68, 1.12)	0.89 (0.69, 1.15)	0.85 (0.66, 1.09)	0.57
Adjusted OR (95% CI) ^2^		ref	0.75 (0.58, 0.97)	0.86 (0.67, 1.11)	0.90 (0.70, 1.17)	0.86 (0.66, 1.11)	0.68
Adjusted OR 2 (95% CI) ^3^		ref	0.76 (0.59, 0.99)	0.87 (0.67, 1.13)	0.92 (0.71, 1.19)	0.87 (0.67, 1.13)	0.76
Pulses	19.4 [10.1–30.6]						
n with/without AIS		216/270	227/259	237/250	240/246	241/245	
Crude OR (95% CI)		ref	1.10 (0.85, 1.41)	1.19 (0.92, 1.53)	1.22 (0.95, 1.57)	1.23 (0.96, 1.58)	0.07
Adjusted OR (95% CI)		ref	1.07 (0.83, 1.39)	1.13 (0.87, 1.46)	1.16 (0.89, 1.50)	1.20 (0.93, 1.56)	0.13
Adjusted OR 2 (95% CI)		ref	1.09 (0.84, 1.41)	1.11 (0.85, 1.44)	1.19 (0.92, 1.54)	1.18 (0.91, 1.53)	0.15
Fruits	32.7 [15.7–58.4]						
n with/without AIS		226/260	225/261	229/258	241/245	240/246	
Crude OR (95% CI)		ref	0.99 (0.77, 1.28)	1.02 (0.79, 1.31)	1.13 (0.88, 1.46)	1.12 (0.87, 1.44)	0.21
Adjusted OR (95% CI)		ref	0.96 (0.74, 1.24)	0.97 (0.75, 1.26)	1.06 (0.82, 1.37)	1.12 (0.86, 1.45)	0.28
Adjusted OR 2 (95% CI)		ref	1.00 (0.77, 1.30)	0.98 (0.75, 1.27)	1.08 (0.83, 1.40)	1.13 (0.87, 1.46)	0.30
Vegetables (excluding mushrooms)	99.2 [68.6–144]						
n with/without AIS		241/245	206/280	243/244	234/252	237/249	
Crude OR (95% CI)		ref	0.75 (0.58, 0.96)	1.01 (0.79, 1.30)	0.94 (0.73, 1.21)	0.97 (0.75, 1.24)	0.57
Adjusted OR (95% CI)		ref	0.72 (0.56, 0.94)	0.97 (0.75, 1.26)	0.92 (0.71, 1.18)	0.95 (0.74, 1.23)	0.64
Adjusted OR 2 (95% CI)		ref	0.74 (0.57, 0.97)	0.97 (0.75, 1.26)	0.91 (0.71, 1.19)	0.95 (0.73, 1.23)	0.75
Mushrooms	3.8 [1.5–6.4]						
n with/without AIS		225/261	254/232	231/256	225/261	226/260	
Crude OR (95% CI)		ref	1.27 (0.99, 1.63)	1.05 (0.81, 1.35)	1.00 (0.78, 1.29)	1.01 (0.78, 1.30)	0.44
Adjusted OR (95% CI)		ref	1.24 (0.96, 1.61)	1.02 (0.79, 1.32)	0.99 (0.76, 1.28)	0.99 (0.77, 1.29)	0.41
Adjusted OR 2 (95% CI)		ref	1.25 (0.96, 1.61)	1.00 (0.77, 1.30)	0.97 (0.74, 1.25)	0.99 (0.76, 1.29)	0.35
Fish and shellfish	25.5 [17.1–36.5]						
n with/without AIS		236/250	244/242	230/257	225/261	226/260	
Crude OR (95% CI)		ref	1.07 (0.83, 1.37)	0.95 (0.74, 1.22)	0.91 (0.71, 1.18)	0.92 (0.72, 1.19)	0.26
Adjusted OR (95% CI)		ref	1.08 (0.84, 1.40)	0.91 (0.70, 1.17)	0.94 (0.72, 1.21)	0.93 (0.72, 1.21)	0.34
Adjusted OR 2 (95% CI)		ref	1.05 (0.81, 1.36)	0.88 (0.68, 1.14)	0.93 (0.72, 1.21)	0.92 (0.71, 1.19)	0.33
Meat	35.0 [26.1–45.8]						
n with/without AIS		233/253	227/259	241/246	238/248	222/264	
Crude OR (95% CI)		ref	0.95 (0.74, 1.22)	1.06 (0.83, 1.37)	1.04 (0.81, 1.34)	0.91 (0.71, 1.18)	0.75
Adjusted OR (95% CI)		ref	0.96 (0.74, 1.24)	1.06 (0.82, 1.37)	1.07 (0.83, 1.39)	0.90 (0.70, 1.17)	0.74
Adjusted OR 2 (95% CI)		ref	0.94 (0.72, 1.21)	1.05 (0.81, 1.36)	1.07 (0.83, 1.39)	0.91 (0.70, 1.19)	0.88
Dairy products	87.3 [45.8–133]						
n with/without AIS		242/244	228/258	238/249	226/260	227/259	
Crude OR (95% CI)		ref	0.89 (0.69, 1.15)	0.96 (0.75, 1.24)	0.88 (0.68, 1.13)	0.88 (0.69, 1.14)	0.36
Adjusted OR (95% CI)		ref	0.86 (0.66, 1.11)	0.95 (0.73, 1.23)	0.88 (0.68, 1.14)	0.88 (0.68, 1.15)	0.46
Adjusted OR 2 (95% CI)		ref	0.83 (0.64, 1.08)	0.94 (0.72, 1.22)	0.89 (0.69, 1.16)	0.87 (0.67, 1.13)	0.48
Tea and coffee	211 [88.1–323]						
n with/without AIS		236/250	240/246	236/251	227/259	222/264	
Crude OR (95% CI)		ref	1.03 (0.80, 1.33)	1.00 (0.78, 1.28)	0.93 (0.72, 1.19)	0.89 (0.69, 1.15)	0.24
Adjusted OR (95% CI)		ref	1.03 (0.80, 1.33)	0.92 (0.71, 1.19)	0.89 (0.69, 1.15)	0.89 (0.68, 1.15)	0.19
Adjusted OR 2 (95% CI)		ref	1.02 (0.78, 1.32)	0.93 (0.72, 1.21)	0.90 (0.69, 1.16)	0.91 (0.70, 1.19)	0.30

IQR, interquartile range; OR, odds ratio; CI, confidence interval. ^1^ AIS was defined as a Cobb angle of ≥15°. ^2^ Adjusted OR was calculated by logistic regression models including age, menarcheal status, school type (public or private), and family composition (single-parent, 2-parent, or 3-generation family) as covariates. ^3^ Adjusted OR 2 was calculated by logistic regression models including classic ballet experience (yes or no), mother’s history of scoliosis (yes or no), use of dental braces (yes or no), and BMI (<18.5, 18.5 ≤ 25, 25≤), as well as the four covariates mentioned above (2). ^4^ Trends of association were tested using logistic regression models, which assigned scores at the level of the independent variable (food intake).
